# 

**Published:** 1807-02

**Authors:** 


### NEW MEDICAL PUBLICATIONS.

An Account of the Ophthalmia, which has appeared in England since the return of the British Army from Egypt. By John Vetch, M.D. 6s. boards.

Practical Observations on the Uterine Hemorrhage, with Remarks on the Management of the Placenta. By John Burns, 5s. boards.

### TO CORRESPONDENTS.

Communications are received from Mr. Carmichael, Mr. Laurence, Mr. J. B. Davis, Mr. Soden, Mr. Dawson, Mr. Harrup, Dr. Huggan, Mr. Atkinson; and we should be glad to receive from all our Correspondents, authentic Cases of Hydrophobia.

